# A Missing Voice: The Lingual Complex and Osteopathic Manual Medicine in the Context of Five Osteopathic Models

**DOI:** 10.7759/cureus.18658

**Published:** 2021-10-11

**Authors:** Bruno Bordoni, Allan R Escher

**Affiliations:** 1 Physical Medicine and Rehabilitation, Don Carlo Gnocchi Foundation, Milan, ITA; 2 Anesthesiology/Pain Medicine, H. Lee Moffitt Cancer Center and Research Institute, Tampa, USA

**Keywords:** cancer, manual therapy, osteopathy, diaphragm, fascia, tongue, osteopathic

## Abstract

The five osteopathic models recognized by the American Association of Colleges of Osteopathic Medicine guide clinicians in the evaluation and therapeutic choice which must be the most appropriate concerning the patient’s needs. Skeletal muscles represent an important interpretation, such as screening and treatment, on which these models are based. A muscle district that is not considered by the usual osteopathic practice is the tongue. The lingual complex has numerous functions, both local and systemic; it can adapt negatively in the presence of pathology, just as it can influence the body system in a non-physiological manner if it is a source of dysfunctions. This paper, the first of its kind in the panorama of scientific literature, briefly reviews the anatomy and neurophysiology of the tongue, trying to highlight the logic and the need to insert this muscle in the context of the five osteopathic models. The clinician’s goal is to restore the patient’s homeostasis, and we believe that this task is more concrete if the patient is approached after understanding all the contractile districts, including the tongue.

## Introduction and background

Osteopathic medicine (OM) was born from the reflections and studies of Andrew Taylor Still, DO, at the end of the 19th century [1]. One of the fundamental principles of OM, in the words of the founder, is: “Osteopathy is based on the perfection of Nature’s work” [2]. The study of anatomy and physiology allows us to understand how to place our hands on the patient, respecting the ability of the human body to perform at its best the functions for which health can be expressed. Following the thought of John Martin Littlejohn, MD DO, OM is an ever-evolving discipline, reflecting advancing scientific knowledge [1]. This means that clinicians must integrate into their daily lives, whenever possible, the practical and theoretical information that constantly emerges from the scientific literature. The ultimate goal is to achieve the patient’s well-being through more conscious ways on the part of the osteopath [3]. To better frame the patient, OM provides five osteopathic models, which are never a constraint but an absolute integration of the different aspects that represent the individual [2]. These models include the biomechanical-structural model, metabolic-energy (nutritional) model, neurological model, respiratory-circulatory model, and behavioral-biopsychosocial model [4]. These models were defined in 1987 by the American Association of Colleges of Osteopathic Medicine (AACOM) Educational Council on Osteopathic Principles (ECOP) and approved by the Council of Deans [2]. In 2012, the Osteopathic International Alliance (OIA) accepted such models as an OM approach used globally [1,2]. The five models help the clinician to observe the patient considering multiple aspects, influencing the subjective manual approach and the choice of the technique used. Each treatment involves all the different components that make up the human body, from the psyche to the spirit, to all the structures (solid and fluid) in motion [2]. The main interface used to access clinical information is the musculoskeletal system, a mirror to look at the patient [2]. The biomechanical-structural model emphasizes the structural aspect of the body, which allows the movement and maintenance of a position (static or dynamic) [2]. Dysfunction of the structure could lead to limitations of movement and independence of the person, visceral alterations (somato-visceral reflexes), and emotional disturbances related to decreased mobility [5]. The metabolic-energy (nutritional) model bases its principles on energy and metabolic homeostasis concerning the energy consumed, both produced and available. The osteopath evaluates the functional capacity of the entire body system, whereas the musculoskeletal system plays a major role in the treatment strategy [2,6]. The neurological model touches on other concepts for the patient’s health concerning the balance connected to the nervous system (central and peripheral, sympathetic and parasympathetic, enteric) [2]. The treatment of skeletal muscle helps restore the most adequate responses related to the proprioception and different visceral functions (improving visceral reflexes), as well as rebalance the mechanisms connected to pain sensations [2,7]. The respiratory-circulatory model orients the clinician on the body’s ability to circulate the different fluids and the multiple information transported, providing nutrients and oxygen to all cells [2]. A muscle district that profoundly affects circulation and respiration, as well as a target of medicine osteopathic manual, is the diaphragm [8]. The behavioral-biopsychosocial model takes into consideration how the patient psychically and physically deals with the environment in which he/she lives; evaluating and treating the musculoskeletal system can be key to understanding how to stimulate the correct salutogenic responses [2,9]. This model helps the patient to become more aware of his own body and emotions, trying to improve behavioral habits [10]. A muscle area that is difficult to evaluate and handled manually by the osteopathic clinician is the tongue. Tongue and its systemic correlations are missing in the most well-known text in the osteopathic world, that is, Foundations of Osteopathic Medicine (2018), just as only three scientific publications consider a manual work of osteopathy on the lingual complex [2,11-13]. This article briefly reviews the anatomy and neurophysiology of the tongue to highlight the logic and the need to insert this muscle in the context of the five osteopathic models. The clinician’s ability to stimulate a physiological and healthy environment for the patient is only strengthened if the tongue is also considered in the evaluation and manual osteopathic treatment.

## Review

Lingual origin and functional anatomy

**Figure 1:**
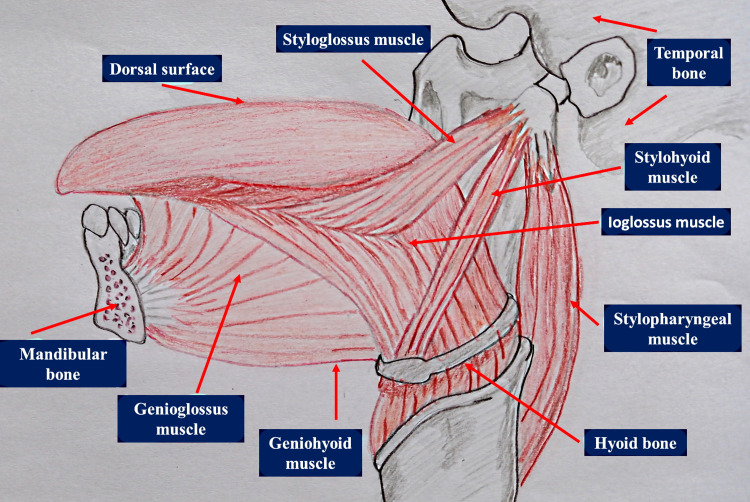
The anatomy of the lingual complex (extrinsic musculature). The figure has been created by Bordoni Bruno.

The branchial arches or pharyngeal arches are metamerized embryonic structures that appear in the cephalic region of the embryo. The viscerocranium originates from the branchial arches.They are formed by the three embryonic sheets, namely, ectoderm, mesoderm, and endoderm [14]. The connective tissue that separates and connects the various muscle bundles that make up the tongue derives from the ectodermal neural crests (dorsal area of the neural tube), as well as the vascular and lymphatic, while the contractile tissue originates from the mesoderm (occipital somites) [15-17]. The movement of the tongue begins in the third month of pregnancy (approximately day 75) with swallowing and in conjunction with flexion of the head and fingers toward the lips [18]. Swallowing during the growth period is important for the entry of amniotic fluid and the stimulation of the formation of the kidneys and the excretory system [18]. The muscle complex of the tongue includes extrinsic (important for movement) and intrinsic (important for shape) districts [16]. The pairs of intrinsic muscles without bone attachment form eight muscles: verticalis, transversalis, inferior, and superior longitudinalis [18]. These muscles are intertwined forming a central perpendicular area, which is inserted into a bundle of fibers arranged horizontally. The intrinsic muscles merge with the extrinsic muscles to collaborate synergistically for the functions assigned to the tongue [19]. The fibers of the longitudinalis muscles (inferior and superior) and the fibers of the transversalis muscle (antagonist of the longitudinalis muscles), according to a study in rats, are composed mainly of anaerobic fibers, demonstrating greater strength than the verticalis muscle [20]. Probably, the strength of these fibers (together with the anaerobic fibers of the genioglossus muscle) allows greater pressure of the tongue against the hard palate, an important action during the initial phase of swallowing [20,21]. The pairs of extrinsic muscles, for a total of eight muscles, include palatoglossus, hyoglossus, styloglossus, and genioglossus [16]. Some studies have considered two other muscles as extrinsic districts of the tongue: glossopharyngeus and chondroglossus [16,18]. The palatoglossus influences the tongue (shape and position) with upward force vectors, while the styloglossus muscle creates tension toward the posteriority. The hyoglossus and genioglossus muscles, when contracting, develop vectors of force toward the inferiority [19]. Styloglossus arises from the styloid process of the temporal bone up to the lingual septum (tip of the tongue), merging with the inferior longitudinal muscle [19]. The muscle acts as a retrograde force of the tongue to propel the food bolus and to better manage small lingual movements during speech [19]. The styloglossus with its contraction can influence the orientation of the intrinsic fibers and the fibers of the extrinsic muscles located in the posterior and lower area of the tongue; this function has not yet been explained [19]. The hyoglossus muscle involves the body and large horns of the hyoid bone (with some fibers it involves the epiglottis and median glossoepiglottic ligament) and merges with the fibers of the intrinsic inferior longitudinalis muscle and the styloglossus muscle [18,19,22,23]. Hyoglossus is important for the stages of swallowing when the epiglottic needs to move for the passage of the bolus [24]. When the palatoglossus muscle contracts, it can pull the soft palate toward the tongue (posterior area) to facilitate the different phases of swallowing, in particular, by pushing the bolus toward the esophagus and preventing the bolus from returning. The two muscular bellies form ridges in the lateral pharyngeal area, defined as palatoglossal arches, thus separating the oral cavity from the oropharyngeal cavity [25]. The palatoglossus also plays the role of an antagonist of the levator veli palatini muscle [25]. It has few muscle fibers and arises from the palatine aponeurosis to insert posteriorly to the lingual body and merging with the intrinsic transversalis and superior longitudinalis muscles [19,25]. The genioglossus muscle involves the superior mental spines and the hyoid bone and constitutes a large portion of the lingual body, in particular, the posteroinferior area; it merges with the verticalis muscle in the anterior portion of the tongue [19,23]. The genioglossus plays important roles in tongue movement, swallowing, speech, and respiration [23]. The genioglossus helps to give a certain shape to the bolus and to push the bolus toward the esophagus through the pharynx [26]. During the act of inhalation, the muscle undergoes an anterior movement in its lower and posterior portion and, at the same time, its posterior and upper portion creates a movement with a caudal and posterior vector; in this way, it moves the pharynx forward and opens the upper respiratory tract [8]. The genioglossus facilitates the protrusion of the tongue during speech (together with the verticalis and transversalis muscles), becoming an agonist of the styloglossus muscle and the hyoglossus muscle (together with the inferior muscles and superior longitudinalis), the latter of which tend to bring the lingual complex into retrusion (Figure 1) [27]. According to some studies, the chondroglossus muscle is synonymous with the hyoglossus muscle, while according to others, it is an individual muscle district [19,23]. Probably, having the same phylogenetic origin, it can be considered an extrinsic muscle of the tongue, or a part of the hyoglossus muscle [19,23]. The glossopharyngeus extrinsic muscle derives from the superior pharyngeal constrictor muscle, to fuse with the intrinsic musculature of the tongue, posteriorly [16,22]. The tongue, through a fold of connective tissue known as the lingual frenum, which is placed under the ventral surface of the tongue in a median position, connects to the buccal floor [28]. The innervation of the tongue is very complex. The glossopharyngeal nerve or the ninth cranial nerve ensures the gustatory capacity for the posterior third of the lingual mucosa and supplies parasympathetic fibers [29]. The ninth anastomosis within the tongue with the cranial nerve X or vagus nerve, with the lingual nerve (branch of the trigeminal nerve or V) and with branches of the XII or hypoglossal cranial nerve [13,16]. The latter is responsible for the motility of the lingual muscles, while the X nerve directly involves the motility of the palatoglossus muscle [30,31]. The lingual nerve innervates the mucous membrane of two-thirds of the anterior area of the tongue, providing somatic and gustatory sensitivity; the nerve is anastomosed in the anterior lingual area with the sensitive fibers of the facial nerve or VII cranial nerve (cord of the eardrum), providing afferent sensitive fibers [32]. Sympathetic fibers from the superior cervical ganglion reach the tongue; this connection possibly regulates the movement of the tongue based on the oxygen present and other functions not yet fully understood [33,34].

The biomechanical-structural model and the tongue

An incorrect position of a muscle area limits the physiological movement of body fluids, negatively impacting the various local and systemic neurological and immunoendocrine responses; over time, this somatic dysfunction could lead to problems related to the psychic sphere, such as depression or anxiety [2]. The patient’s adaptive capacity and homeostasis are impacted [2]. The osteopath will have to evaluate the dysfunctional somatic district and decide which manual strategy to apply, with the goal of eliminating the cause of this dysfunction. The tongue is a target for evaluation and manual treatment in the clinical process of the osteopath, despite being a concept blatantly absent in the main text of the osteopathy world [2]. The text in question (Foundations of Osteopathic Medicine. Philosophy, Science, Clinical Applications and Research) reports in the biomechanical-structural model the example of a whiplash, where many dysfunctions, not only local (neck), can negatively affect the entire body system [2]. Remaining in the example of whiplash, we know that traumatic adaptation can lead to negative consequences for lingual functions, probably due to the muscular and neurological connections with the cervical tract, thoracic outlet, and hyoid bone [11]. The cervical tract and the head in the presence of alterations in the flexion-extension capacity (as in the example of whiplash) can negatively affect the movement of the temporomandibular joint; mandibular dysfunction alters the position and function of the tongue [11,18,35]. The same body posture affects the ability of the tongue to generate sufficient pressure for adequate swallowing; the latter action is also influenced by the movement of the jaw and the hyoid bone in a perfect muscular-articular balance of the stomatognathic and craniocervical system [21,36,37]. A dysfunctional muscular area can cause local or distant dysfunction (“vicious cycle theory”), and consequently, a lingual complex with functional limits can cause somatic symptoms of the cervical tract through the muscular connections of the oropharyngeal, cervicomandibular, and thoracic-hyoid tract [11,18,38,39]. The same theory of myofascial chains poses interesting scenarios for framing with a renewed gaze the clinical picture between the tongue and apparently distant areas owing to a continuous myofascial network [40]. Remaining in the example of trauma in the car, a reflex shot to the face could break some teeth; the reduced number of teeth (and advancing age) leads to a reduction in the force expressed by the tongue in conjunction with a decreased strength of the skeletal muscles and a reduction in functional independence [41]. The tongue has a systemic effect as it forms an integral part of the body system [16]. Like any muscle district, the tongue can increase its volume (hypertrophy) or decrease its diameter (hypotrophy) depending on the stimuli present, both physiological and non-physiological; as with other skeletal muscles, specific lingual exercises improve physiological functions [42-44]. With advancing age, lingual muscle fibers undergo apoptosis and the number of contractile fibers reduces.

The metabolic-energy (nutritional) model and the tongue

**Figure 2:**
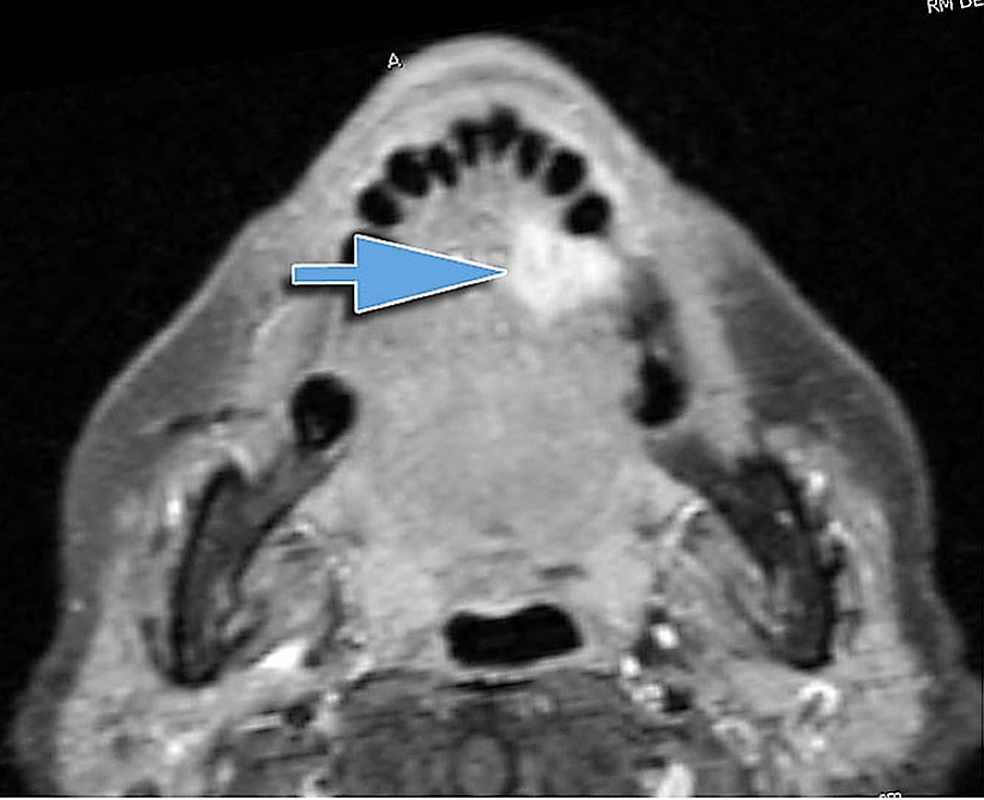
Squamous cell carcinoma of the left tongue. The MRI shows an extension in depth greater than what is visible. The neoplasm approaches very close to the midline (arrow) but does not reach it. MRI: magnetic resonance imaging

The energy introduced must be optimal with respect to the body’s energy expenditure; a muscle dysfunction could alter the metabolic homeostasis and reduce the patient’s adaptive capacity [2]. Improving the function of the skeletal muscle area subject to dysfunction increases the endocrine, fluidic and immune skills, local and systemic, with the ultimate aim of restoring the functional independence of the subject [2]. The tongue plays a fundamental role in the nutritional supply process through the stages of swallowing. We can distinguish three phases in swallowing depending on the anatomical area where the bolus is present: oral, pharyngeal, and esophageal [45]. In the oral phase, the tongue with the bolus (solid or fluid) moves away from the soft palate bringing the apex toward the retroincisal-palatal spot, and it touches and presses against the hard palate and pushes the bolus posteriorly toward the pharynx (pharyngeal phase) to finally enter the bolus into the esophagus (esophageal phase); after this last phase, the tongue resumes contact with the soft palate [28,46]. The portions of the tongue that move the most (elevation) during these phases are the frontal, medial, and posterior areas; in particular, the posterior portion rises more [46]. During swallowing, the tongue (lower lingual portion) moves the hyoid bone in horizontal and vertical actions; in the pharyngeal phase, the hyoid is pushed forward and upward [45]. Lingual dysfunctions, attributable to advancing age (very frequent) or to the presence of disorders and pathologies, cause dysphagia which decreases the patient’s optimal nutrition [47,48]. Another fundamental aspect of the tongue for the model is related to taste. The sense of taste is considered the most rostral way of the enteric system; the activation of the trigeminal lingual afferents thanks to the different food components can stimulate the digestive functions before the food arrives in the gastrointestinal system [49]. Depending on the chemical components of the food, the taste allows to specifically stimulate the enzymes more suitable for food, preparing the digestive system and systemic metabolism in advance [49,50]. The same type of diet can produce changes in the ability of taste to decipher the different chemical components, as well as negatively or positively influence digestive behavior [51]. A chronic disease, from cancer to a systemic disease, can alter the activity of taste receptors, in addition to motor function. This event could disturb metabolic homeostasis and cause and obesity (Figure 2) [51,52].

A decrease in the muscle strength of the tongue is a prelude to the advent of possible local and systemic dysfunctions [53]. In the reference book of osteopathy, the example of a pathology on which the clinical reasoning that fits into the model is built is chronic heart failure (CHF) [2]. Patients with CHF often present with obstructive sleep apnea syndrome (OSAS) [54]. We know that one of the main causes of OSAS is morphological and functional alterations of the tongue, in particular, an increase in posterior volume and an alteration of the intervention of the autonomic nervous system [55,56]. Lingual dysfunction in CHF and OSAS raises the likelihood of systemic metabolic disorders, with the risk of further cardiovascular sequelae, the onset of metabolic syndrome, and cognitive impairment [56]. Tongue assessment and manual treatment reflect the criteria of the metabolic-energy (nutritional) model.

The neurological model and the tongue

**Figure 3:**
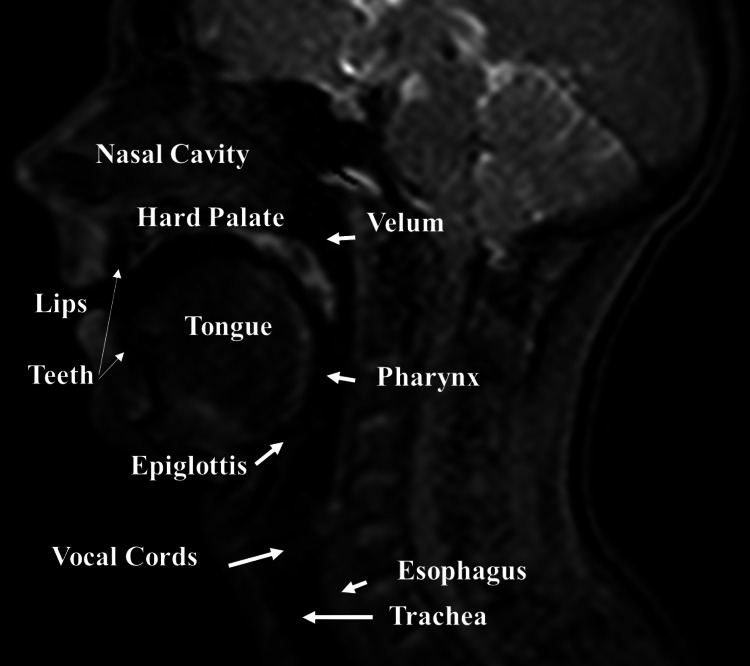
Midsagittal slice of cine magnetic resonance - MRI. The figure illustrates some areas of the skull and their position, such as the tongue, nasal cavity, pharynx, and epiglottis.

An alteration of the neurological pathways can be expressed with somatic dysfunction, which leads to a decrease in the capacity of the immune-endocrine and circulatory system, and with negative repercussions on the behavioral sphere [2]. Homeostasis undergoes a decline, with less capacity to adapt. Muscle districts tend to lose proprioception and neuromotor coordination, lowering the threshold of pain-related responses [2]. In this model, some mechanisms related to symptomatological response are cited in the book by Seffinger, DO, such as spinal facilitation deriving from non-muscular structures only [2]. An osteopathic strategy is to work the spinal muscles and improve vertebral movement to interrupt the non-physiological closed circuit that has arisen, which determines the symptom [2]. The tongue has a precise somatotropic representation at the cortical level, with numerous connections with the limbic area, the midbrain, and medulla oblongata, as well as the cerebellum and vestibular area [16]. The position of the tongue affects the central nervous system. To give an example, the posterior and lateral movements of the lingual body activate the anterior cingulate cortex, which is activated for the management of pain information, the emotional and cognitive sphere, as well as to process visceral and sensitive sensations [16]. Through connections with the pharyngeal plexus and the ansa cervicalis, the innervation of the tongue is related to the peripheral neurological area of the occipitocervical tract [57,58]. The tongue is richly structured with mechanoreceptors and proprioceptors (corpuscles similar to those of Meissner, and corpuscles of Krause) to discern the mechanical sensations in the presence of solid structures (bolus, saliva, teeth) and movements of the lingual musculature, with sensitivity equal to that of touch [59]. We can find other types of corpuscles linked to the sensation of pain or nociceptors through unmyelinated afferents [59]. With advancing age, lingual mechanical-sensory abilities undergo a decline in the functional quality of the tongue (swallowing, phonesis, breathing) [59]. With advancing age, the activation threshold of the lingual muscles increases, with activation delays and non-coordination of the tongue. The position of the tongue influences posture and peripheral muscle function through neurological connections. The electrical neurostimulation of the tongue by external sensors for patients with sensory disorders related to pathologies of the cerebellum and vestibular area can correct body movement, helping the person to walk independently [60-62]. The tongue represents an important interface with the nervous system [63]. The position of the tongue within the oral cavity determines the expression of the muscular performance of the limbs; when held against the upper retroincisal-palatal spot during muscle activity, strength increases by approximately 30% in healthy individuals [64]. Probably, lingual afferents and trigeminal afferents located in the palatal spot are stimulated [16,63]. There is greater coordination of skeletal muscles when the tongue presses against the palatine spot, improving the postural aspect (in healthy individuals) [65]. The active and repetitive movements of the tongue improve the information that reaches the autonomic system (through afferents of the vagus nerve and the sympathetic system), with an efferent increase in the parasympathetic system, lowering the production of stress hormones [66,67]. The tongue has the full right to re-enter this osteopathic model and to be evaluated and possibly treated (Figure 3).

In the reference book of osteopathic medicine, an example is given of altered systemic reflexes following surgery, which can be felt through palpation of the spinal musculature showing rigid muscles and the limitation of vertebral movement [2]. After an event, surgery, for example, in the cardiovascular field, patients present with problems in lingual function, with the presence of dysphagia [68,69]. Surgery to remove tumor masses from the neck or head generally favors the onset of dysphagia [70]. The cervical area in patients with dysphagia is stiff, painful, and with limitation of vertebral movement; the craniocervical posture influences the function of the tongue [35,71-73]. There are prerequisites for palpating the craniocervical area to assess the possible presence of lingual dysfunction, as well as manual indications to improve the same lingual functions [71,74,75].

The respiratory-circulatory model and the tongue

This model considers the proper functioning of the breath and the possibility of an optimal circulation of body fluids to be fundamental in trying to obtain a cellular environment (intra and extra) in full efficiency [2]. The supply of nutrients and the removal of excess metabolites can be achieved by creating freedom of action for the different components of the body or by limiting (psychic) attitudes or harmful habits (nutrition) [2]. The usual evaluation strategy and the possible treatment are oriented toward the musculoskeletal areas [2]. The tongue is a key muscle of breathing. The central pattern generator (CPG) is responsible for respiratory actions, located in the brainstem, bulb, and spinal cord; the first muscle that contracts and is managed by the CPG is the lingual complex [8]. The neurons of the preBötzinger complex (brainstem) stimulate the activity of the XII cranial nerve, thus opening the pharyngeal space or retroglossal airway; the preBötzinger complex continues its afferent action to start activating the phrenic pre-neurons and the contraction of the diaphragm muscle and the inhalation [8]. Another neural area defined as the post-inspiratory complex near the bulb, in case of the need to prolong inhalation (cough, phonesis, swallowing), can help stimulate the electrical activity of the XII cranial nerve (and the vagus nerve), lengthening the involvement of the tongue (and the muscles of the larynx) [8]. The expiratory phases to inhibit lingual contraction involve other brain areas of the brainstem, such as the Bötzinger neurons, the parabrachial/Kölliker-Fuse complex, and the retrotrapezoid nucleus [8]. Dysfunction of the tongue leads to decreased quality of breath, for example, with the onset of sleep apnea or OSAS, not allowing adequate opening of the pharyngeal space [76]. This pathological situation causes insomnia, cognitive decline and possible depression, and cardiovascular pathologies [76]. The functional situation of the tongue reflects the muscle strength of the limbs, as well as neuromuscular coordination (walking), placing lingual adaptation in a systemic context. A lingual functional decline coincides with a lower strength expressed (handgrip strength) in elderly individuals with a decrease in walking speed [41,77]. It is not possible to obtain a global vision of the patient’s homeostatic behavior, without also taking into account the tongue. The tongue is rich in lymphatic passages (in addition to the vascular pathways), starting from the mucous membrane of the tongue up to the neck and submental area; the tongue contains several lymphatic nodes, which are found within the lymphatic ducts [78]. The lymphatic nodes are found mainly in the anterolateral and posterolateral lingual areas within the musculature [78]. The marginal lymphatic vessels drain the lymph from the mucosa to the submucosal area from the anterior region of the tongue up to the submental and submandibular lymphatic nodes [78]. The posterior marginal lymphatic pathways reach the lymphatic nodes of the cervical tract; the central ducts drain up to the neck and submental nodes [78]. The lingual lymphatic ducts also collect lymph from the tissues of the oral mucosa; the density of the lingual lymphatic vessels increases moving from the tip of the tongue to the base, as well as the diameter of the vessels themselves [78,79]. Muscles are important structures for stimulating the passage of lymphatic fluids [78,79]. The osteopathic reference book, Foundations of Osteopathic Medicine, gives the example of pneumonia, in which congestion of body fluids and dysfunction of the diaphragm muscle can be found [2]. Pneumonia can result from unwanted aspirations due to the presence of dysphagia, an event that has a high percentage in the elderly [80]. Lingual dysfunction may occur following a prolonged position with the patient prone, following pneumonia due to severe acute respiratory syndrome coronavirus 2 infection; lingual functional impairment can have serious consequences for the patient’s health [81]. Similarly, with chronic pulmonary diseases such as chronic obstructive pulmonary disease and asthma, there are abnormal lingual movements, a functional decline in the strength and activity of the tongue, which reflects the decline in muscle strength of the limbs [82-84].

The behavioral-biopsychosocial model and the tongue

This model is based on the main evaluation of the psychophysical and spiritual well-being of the patient through the investigation of the environment (cultural and socioeconomic) in which the individual lives and through a musculoskeletal evaluation, which can present dysfunctions reflecting a biopsychosocial disorder [2]. Stress derived from external factors can lead to non-physiological adaptations of the body’s muscular system; the clinician’s goal is to restore the maximum freedom of movement to improve the patient’s adaptive capacity [2]. The position and behavior of the tongue reflect emotional states. Emotions linked to fear are expressed by the tongue with the tip held more downwards, that is, towards the soft palate [85]. Negative sensations related to morality (violation of one’s morality) tend to decrease the cortical motor areas connected to the tongue (in particular, the M1 area), decreasing the functions expressed by the lingual complex [86]. Psychiatric disorders (chronic anxiety and stress), as in the presence of post-traumatic stress disorder, lead to an alteration of the function of the XII nerve and the lingual position, with greater evidence of obstructive sleep apnea [87]. The production of phonemes and the movements of the tongue can be conditioned according to what we observe (good or bad events), with an activation of the cortical area involved in lingual motor skills [88]. Patients who suffer from major depression for several reasons have a reduction in the activity of the vagus nerve; the latter innervates a muscular portion of the tongue (palatoglossus), negatively affecting the lingual motor function [89]. The palatoglossus muscle plays an important role in the production of phonesis through movement in elevation and retrusion, in particular, in the management of an anatomical area essential for speech (oropharyngeal isthmus); this last anatomical region separates the oral and pharyngeal spaces of the vocal tract [90]. A reduction in the ability to form words, with decreased muscle tone and a decline in communicating with other people, worsens patients’ quality of life [91]. In the book on osteopathy, which represents a global reference for osteopathic clinicians (Foundations of Osteopathic Medicine), the example of a patient with emphysema is presented for this fifth model and the consequences that this pathology causes from respiratory problems to psychic dysfunctions [2]. Emphysema, together with chronic bronchitis, is a phenotype that falls within chronic obstructive pulmonary disease (COPD) [92]. We know that COPD leads to neuromuscular non-coordination of the tongue, which is expressed as dysphagia and/or obstructive sleep apnea; the constant respiratory dysfunction with unintentional pulmonary aspirations, nocturnal hypoxia, and continuous awakenings lead to a cognitive decline with psychiatric alterations (anxiety and depression) in patients suffering from these disorders [82,93,94]. We can affirm that a function of the tongue non-optimally can push the patient toward a non-physiological psychiatric sphere, where anxiety and depression cause decreased quality of life of the person [95]. The tongue is part of the behavioral-biopsychosocial model and should be routinely evaluated and possibly treated by the osteopath.

Final reflections

A focal concept that distinguishes osteopathic practice and the clinic is the consideration of the patient as a unit, where homeostasis is in equilibrium when all its parts are in harmony [2]. Leaving the tongue out of the usual osteopathic approach clashes with this philosophy. The tongue is an integral part of the body system; its functional alteration can be expressed with weakness of the trunk muscles and cause back pain [96]. This event can probably be linked to the myofascial and neurological relationships that affect the lingual complex and the rest of the body [16,57,97]. The pelvic floor muscle contractions involve not only the neurological areas connected to these districts but also involves the brain areas corresponding to the activation of the tongue muscles, such as the M1 area, without overlapping. This implies that the tongue maintains its own cortical identity and that it is stimulated independently of its functions of phonesis, swallowing, and breathing, as well as for the musculoskeletal movement of the body [98]. The movement of the tongue during sounds emitted from the mouth is governed by the motor areas, but, at the same time, the limbic areas are always activated, tying together the lingual movement and emotions [99]. The tongue as a skeletal muscle deserves to be evaluated when there are disorders of the psychic sphere. The literature is rich in demonstrating the lingual systemic relationships and the body system. The increase in human survival age or aging produces multiple musculoskeletal, metabolic, nutritional, immune, and psychic disorders, and, like all muscle groups, the tongue undergoes adaptations linked to advancing age, contributing to the finding of altered homeostasis [100-102]. With aging, fat increases (in particular, in the posterior area) along with sarcopenia of the tongue muscles, which scenario negatively affect all lingual functions [102-104]. Considering that chronic problems are more easily found in the elderly, it becomes necessary to insert the tongue for evaluation and possible treatment [105]. In an animal model, chemoradiation revealed a deterioration in lingual function, with the appearance of apneas and non-physiological swallowing. The osteopath should use the art of palpation (and treatment) for the tongue (in addition to the connected areas such as the hyoid bone, the jaw, the occipitocervical area). We hope that this article can be a stimulating starting point for further insights into the osteopathic field and the lingual system and to find a section dedicated to this muscle in the next edition of the book “Foundations of Osteopathic Medicine” [2]. In the literature there is already a text describing how to touch the tongue, but further efforts need to be made to improve the work of the osteopath [13]. To help discriminate the functions of the lingual complex, a single non-instrumental text exists in the scientific literature, which describes the Performance Tongue Test [106]. The latter test can help the clinician to highlight the possible dysfunctions with respect to the presence of other symptoms. The results will be further investigated, if necessary, with instrumental examinations.

## Conclusions

The article sought to lay the foundations to highlight the need to include the lingual muscle complex in the usual evaluation (and possible treatment) by osteopathic clinicians, as it is currently lacking in the global scientific panorama. The text briefly reviewed the functional anatomy of the tongue, trying to associate the five existing osteopathic models, which are a way of framing the patient and which represent the philosophy of osteopathy (the body unit), with lingual functions and dysfunctions. Recognizing the lingual muscle as an anatomical district that must fall within the subdivision and classification of the models would allow us to have a global view of the patient, without neglecting a contractile district so fundamental for homeostasis. This article is the first scientific paper that pushes the osteopath toward a profound reflection, that is, to not lose the art of palpation and the manual approach to the tongue.
